# Identification of FDFT1 as a potential biomarker associated with ferroptosis in ccRCC

**DOI:** 10.1002/cam4.4716

**Published:** 2022-03-24

**Authors:** Ruizhen Huang, Chiyu Zhang, Xing Wang, Xin Zou, Zhengjie Xiang, Zewei Wang, Bin Gui, Tao Lin, Honglin Hu

**Affiliations:** ^1^ Department of Urology The Second Affiliated Hospital of Nanchang University Nanchang China; ^2^ Jiangxi Province Key Laboratory of Molecular Medicine Nanchang China

**Keywords:** biomarker, FDFT1, ferroptosis, renal cancer

## Abstract

Renal cell carcinoma (RCC) seriously threatens people's lives and health. The identification of some precise biomarkers during the process of RCC progression and the pathophysiologic procedure is critical for improving the diagnosis and management of RCC. Evidence suggests that ferroptosis may play a pivotal role in eradicating clear cell RCC (ccRCC, KIRC) tumor cells. We screened out the target prognostic ferroptosis‐associated genes and examined the functions of farnesyl‐diphosphate farnesyltransferase (FDFT1) in 786‐O cells by plasmid transfection. In our study, we identified FDFT1 as a potential marker correlating with ferroptosis in KIRC. Upregulated FDFT1 inhibited cell proliferation, migration, and invasion, and the underlying antitumor effects may occur via the AKT signaling pathway. Our study provides helpful evidence to study the complex physiopathology of KIRC.

## Graphical abstract

By regulating the farnesyl‐diphosphate farnesyltransferase (FDFT1) expression, GPX4 expression, iron, and malondialdehyde levels were significantly changed, FDFT1 inhibited KIRC cell proliferation, migration, and invasion may be associated with ferroptosis.

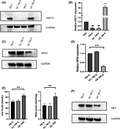

## 1 INTRODUCTION

Renal cell carcinoma (RCC) is a genitourinary system tumor that seriously threatens people's lives and health. The complex pathophysiological process of RCC, which makes up approximately 16% of patients, is metastatic at the time of diagnosis and has a low 5‐year relative survival rate, resulting in more than 65,000 new cases being diagnosed and almost 15,000 deaths every year in the United States.^1^ There are three major histological subtypes of RCC, including clear cell RCC (ccRCC), papillary RCC (pRCC), and chromophobe RCC (chRCC). Among these three histological subtypes, ccRCC accounts for 70%–80% of RCC cases.^2^ Surgical resection is the primary recommended therapeutic method for localized ccRCC; however, poor prognosis was also reported; approximately 30%–40% of ccRCC patients initially diagnosed with advanced‐stage disease developed metastatic recurrence, with 50%, 30%, and less than 11.2% survival rates at 1, 3, and 5 years, respectively, according to recent reports.^3^, ^4^ Herein, a comprehensive understanding of RCC is paramount to the development of improved patient management and treatment. In particular, the identification of some precise biomarkers during the process of RCC progression and pathophysiologic procedures is critical for improving the diagnosis and management of RCC.

Thanks for the considerable development in transcriptome profiling, providing robust data that allow researchers to download cancer data for comprehensive analysis via bioinformatic technology. Furthermore, the application of the bioinformatics method may promote the identification of potential specific markers to encourage the early diagnosis or management of specific malignant tumors. For example, the Cancer Genome Atlas (TCGA) database provides publicly available cancer genomics data, including RNA sequence, copy number variation, DNA methylation, etc., which enables researchers to generate primary analysis before the comprehensive understanding of specific cancers.

Ferroptosis is a novel form of cell death characteristic of iron‐dependent and reactive oxygen species (ROS)‐reliant and was first proposed by Dixon in 2012. In contrast to autophagy and apoptosis, ferroptosis is mainly triggered by iron‐dependent ROS accretion and subsequent extramitochondrial excessive lipid oxidation, which ultimately induces programmed cell death.^5^ Studies have shown that induction of ferroptosis can selectively eliminate a variety of tumor cells, which has been gradually regarded as a promising tumor treatment strategy. Emerging evidence suggests that ferroptosis plays a pivotal role in eradicating tumor cells deficient in key nutrients or damaged by ambient stress.^5^, ^6^, ^7^ Farnesyl‐diphosphate farnesyltransferase 1 (FDFT1, squalene synthase) has been identified as a ferroptosis‐related gene, and it is considered an important gene for prognosis prediction in colorectal cancer patients.^8^, ^9^ However, little is known about its mechanism in tumorigenesis and ferroptosis.

Characterized by uncontrolled cell proliferation and partial resistance to radio‐ and chemotherapies, unsatisfactory outcomes are not uncommon in ccRCC patients. Traditional chemotherapeutic drugs are challenged by poor selectivity, strong side effects, and drug resistance.^10^ Despite great advances, novel therapeutics are still needed to address the poor outcomes in ccRCC. Ferroptosis is a promising strategy in renal cell carcinoma. The relationship between ferroptosis and ccRCC has attracted researchers' attention, and great efforts have been made to illustrate the molecular mechanism of ferroptosis in ccRCC. Yang et al.^11^ conducted a study on the effect of erastin in 60 tumor cell lines of eight tissues, and the representative RCC cell Lines 786‐O and Caki‐1 were used in their study. They found a high sensitivity of RCC cells in erastin‐induced cell death, which in other words, renal cell carcinomas were sensitive to ferroptosis. In addition, ccRCC cells were confirmed to be highly dependent on GSH synthesis in the processes of lipid peroxidation and ferroptosis, and any measures interfering with GSH generation may suppress renal tumor growth and restore normal renal tissue morphology.^12^ Our study was designed to screen some potential ferroptosis‐related genes and verify their role in ccRCC via in vitro experiments. The goal of our study was to elucidate their molecular mechanism and provide a theoretical basis for clinical treatment in ccRCC.

## 2 MATERIALS AND METHODS

### 2.1 Acquisition of ccRCC microarray expression profile

To conduct a comprehensive analysis of differentially expressed genes (DEGs) between ccRCC patients and normal tissues, the primary RNA sequencing (RNA‐seq) data and corresponding clinical information of ccRCC patients were downloaded from the TCGA cancer genomics program (https://portal. gdc.cancer.gov/repository). Then, the gene expression profiles were cleaned, sorted, and analyzed using the scale method provided in the “limma” R package. The data from TCGA are publicly available, and our study follows the TCGA data access policies and publication guidelines. Moreover, 60 ferroptosis‐related genes were obtained from previous studies.^13^ This study was assessed and approved by the Ethics Committee for Animal Experiments of the Second Affiliated Hospital of Nanchang University.

### 2.2 Identification of prognostic ferroptosis‐related genes

The DEGs between ccRCC and non‐cancerous samples were screened initially, adjusted *p* value <0.05 and |log2 FC| > 1 were set as the filter criteria, and all DEGs were selected and identified by using the “edgeR” R package and the “limma” R package. The clinical information and the expression of ferroptosis‐related genes in each sample were combined. Univariate regression analysis was performed to identify genes associated with prognosis, and a *p* value <0.05 was considered statistically significant to screen out prognostic genes. Candidate prognostic ferroptosis‐related genes were the intersection between prognostic genes and differentially expressed ferroptosis‐related genes.

### 2.3 Enrichment analyses

The “clusterProfiler” R package was utilized to conduct Gene Ontology (GO), which was generated to annotate genes as well as identify featured biological properties of transcriptomic profiles and Kyoto Encyclopedia of Genes and Genomes (KEGG) analyses based on the prognostic ferroptosis‐related genes to systematically analyze gene functions. The visualization was performed by the “ggplot2” R package.

### 2.4 Protein–Protein Interaction network and correlation plot analysis

Using the online protein functional interaction database Search Tool for the Retrieval of Interacting Genes (STRING; http://string‐db.org), a protein–protein interaction (PPI) network was built, which may provide the perception of the pathogenesis or progression of diseases. Then, the possible predicted interactions among ferroptosis‐related molecules were indicated by the correlation plot, implemented through the “igraph” and “reshape2” R packages. The most significant modules in the PPI networks were identified using the plug‐in Molecular Complex Detection (MCODE). The selection criteria were as follows: MCODE scores ≥ 3, degree cutoff = 2, node score cutoff = 0.2, max depth = 100, and *k*‐score = 2. The results were regarded as the hub genes for subsequent studies.

### 2.5 Survival analysis

The GEPIA database is a web platform that provides servers for cancer and normal gene expression profiling and interactive analyses.^14^ Based on the TCGA and GTEx gene expression profiles, this platform allows users to perform further survival analysis based on the gene expression levels of the specific selected genes. For example, by inputting a selected gene, a user can overview the overall or disease‐free survival (DFS) in their custom cancer types. We performed survival analysis based on the GEPIA platform and evaluated the relationship between hub gene expression and the survival rate in ccRCC patients.

### 2.6 Cell cultures and cell transfections

786‐O and HK‐2 cells were purchased from the Cell Bank of Shanghai Institutes for Biological Sciences, Chinese Academy of Sciences. 786‐O and HK‐2 cells were cultured in Dulbecco's modified Eagle's medium (DMEM) and minimum essential medium, respectively, containing 1% penicillin–streptomycin and 10% fetal bovine serum (FBS; Gibco) in 50‐mL culture flasks at 37°C under the condition of 5% CO_2_.

The designed FDFT1 overexpression plasmid pcDNA3.1/KLF4‐HisB and the corresponding negative control (NC) plasmid pcDNA3.1‐EF1a‐mcs‐3flag‐CMV‐EGFP (Hanbio) were used for transfection. Cells were seeded into six‐well plates before transfection and were allowed to grow until 70%–80% confluence in each well. For transfection, Lipofectamine 3000 reagent (Invitrogen) was subsequently used to transfect cells with the FDFT1 overexpression plasmid and the corresponding control plasmid according to the manufacturer's instructions. Cells were collected 48 h after transfection for the subsequent studies.

### 2.7 RNA extraction, cDNA synthesis, and RT–qPCR

TransZol Up reagent (TransGen Biotech) was used to extract total cellular RNA. Reverse transcription was performed to synthesize cDNA using the EasyScript® One‐Step gDNA Removal and cDNA Synthesis SuperMix reagent Kit (TransGen Biotech) according to the manufacturer's directions. Then, we used the SYBR Premix Ex Taq kit (Takara) for RT–PCR. Sequences for these genes are shown below: FDFT1 (sense: 5′‐GCAACGCAGTGTGCATATTTT‐3′, antisense: 5′‐CGCCAGTCTGGTTGGTAAAGG‐3′); GAPDH (sense: 5′‐ACCCAGAAGACTGTGGATGG‐3′, antisense: 5′‐TCAGCTCAGGGATGACCTTG‐3′), GPX4 (sense: 5′‐ACAAGAACGGCTGCGTGGTGAA‐3′, antisense: 5′‐GCCACACACTTGTGGAGCTAGA‐3′).

### 2.8 Wound healing assay

To evaluate the function of the FDFT1 gene in cancer cell migration, a wound healing assay was conducted in vitro. Transfected cells, NC group cells, and control group cells were inoculated into six‐well plates, and a wound line was created using a sterilized 200 μl plastic tip to scratch across the surface of cells when they grew to approximately 80%–90% confluence. The plates were washed twice with PBS to remove the suspended cells. The images of 0 h wound closure were photographed. After incubation with reduced serum culture medium for another 24 h, the cell wound closure was photographed again.

### 2.9 Transwell invasion and migration

Transwell assays were performed to examine the cancer cell migration and invasion abilities. A total of 1 × 104,786‐O cells with 100 μl serum‐free DMEM cell suspension were seeded into the upper Transwell chamber, and 600 μl DMEM with 10% FBS was added into the lower chamber for the cell migration assay. For the cell migration analysis, we used Matrigel to mimic the extracellular matrix in the upper Transwell chamber before 1 × 104,786‐O cells were seeded into the surface of Matrigel. The same procedure was used for the migration assay in the bottom chamber. After incubation for 24 h (migration) and 48 h (invasion), the Transwell chambers were removed, and the cells were fixed with methanol. After the application of methyl violet staining solution, the penetrating cells were screened under a light microscope.

### 2.10 Iron assay and lipid peroxidation assay

An iron assay kit (BioAssay Systems) was used to test the ferric iron level. The relative malondialdehyde (MDA) level was evaluated by a lipid peroxidation detection kit (Solarbio, Beijing, China) according to the manufacturer's instructions.

### 2.11 Western blot

We first extracted total protein using RIPA lysis solution, and then a bicinchoninic acid protein determination assay (Solarbio) was implemented to examine the protein concentration. The cell proteins were separated by 10% SDS–PAGE, and the proteins were transferred to PVDF membranes in 1× Western blot transfer buffer. Five percent skimmed milk was used to block the PVDF membrane, and primary FDFT1 antibodies (1:5000; Proteintech), GAPDH antibodies (1:10,000; Proteintech), GPX4 antibodies (1:5000; Proteintech), and AKT antibodies (1:2000; Proteintech) were incubated with the membrane at 4°C overnight. After incubation with the secondary antibody, ECL (US Everbright Inc.) solution was used for further analysis.

### 2.12 EdU assay

To identify the function of FDFT1 in cancer proliferation, an EdU assay was performed. A total of 1 × 104 NC 786‐O cells and FDFT1 overexpression plasmid‐transfected 786‐O cells were plated in a 96‐well plate every well. The next day, the EdU assay was performed according to the manufacturer's instructions (EdU Imaging Kits; US Everbright Inc.). After the application of DAPI dye, cancer cell proliferation was examined by fluorescence microscopy.

### 2.13 Colony formation assay

A total of 500 cells/well were plated into a six‐well plate and incubated at 37°C in a humidified atmosphere with 5% CO_2_. After 14 days, the six‐well plate was removed and subsequently fixed with 4% paraformaldehyde and stained with methyl violet for colony counting.

### 2.14 Statistical analysis

All data were statistically analyzed using GraphPad Prism 8.0 software. Quantitative data are expressed as the mean (*x*) ± S.E. Differences of two groups were compared using the *t* test, and multiple groups were compared using one‐way ANOVA along with the Tukey post hoc multiple‐comparisons test; *p* < 0.05 was considered statistically significant.

## 3 RESULTS

### 3.1 Identification of the target prognostic ferroptosis‐associated genes

**FIGURE 1:**
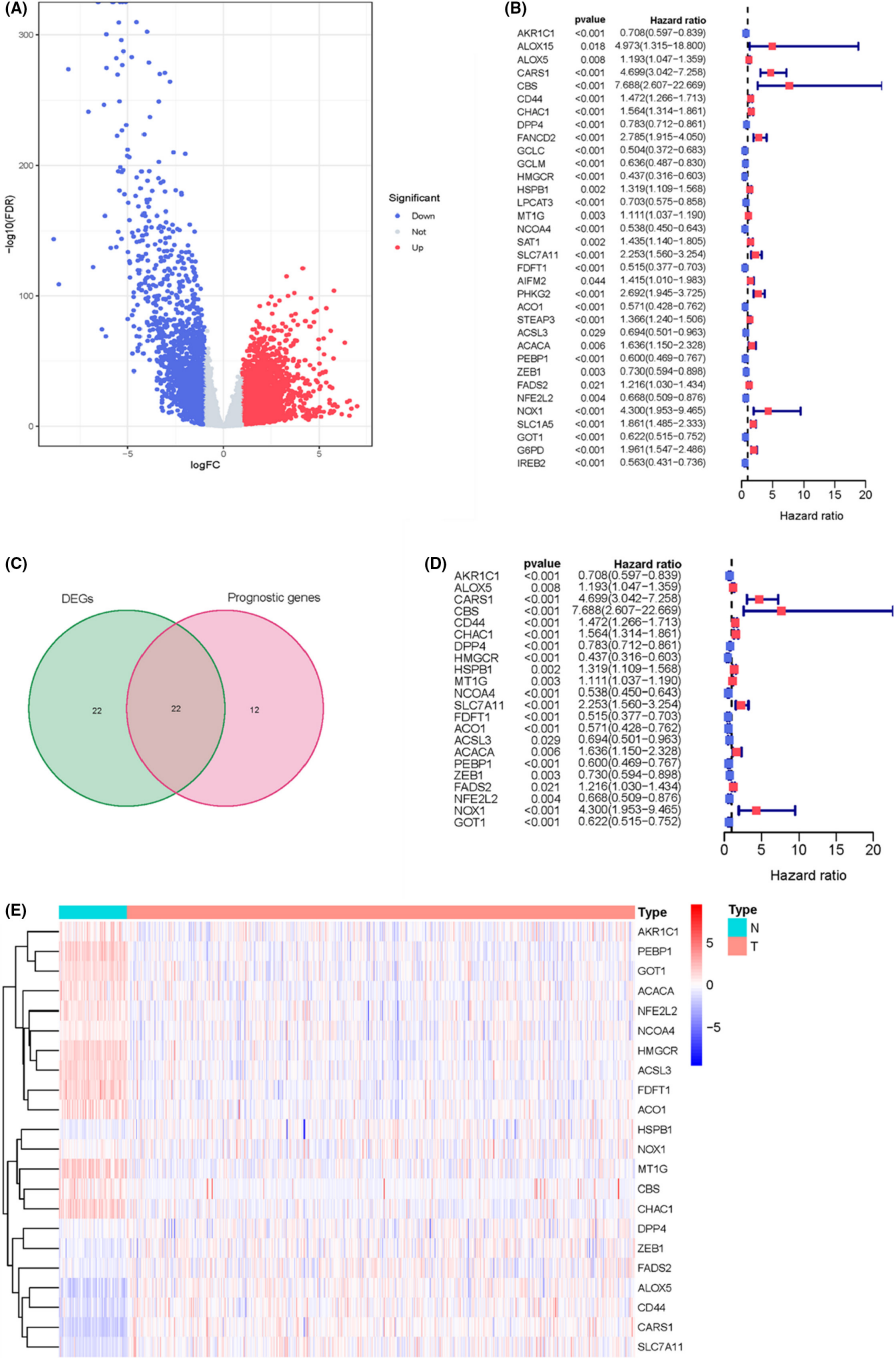
Screening of prognostic ferroptosis‐associated genes. (A) Volcano plot of differentially expressed genes. (B) Prognostic genes. (C) Venn diagram. (D) Prognostic ferroptosis‐associated genes. (E) Heatmap of prognostic ferroptosis‐associated genes

The DEGs between normal tissues and KIRC were visualized by a volcano plot (Figure 1A). The significantly downregulated DEGs are displayed as a blue dot plot, and the red dot plot shows the upregulated DEGs. The prognosis‐related genes were screened by univariate Cox regression analysis based on the expression profile of ferroptosis‐associated genes in each sample (Figure 1B). The prognosis‐related ferroptosis‐associated genes were the intersection between ferroptosis‐associated genes and prognosis‐related genes (Figure 1C). The expression of these genes between normal tissues and tumors was visualized by the heatmap (Figure 1E), as well as the exhibition of prognostic risk among prognostic ferroptosis‐associated genes in the forest plots (Figure 1D).

### 3.2 PPI network and enrichment analyses

**FIGURE 2:**
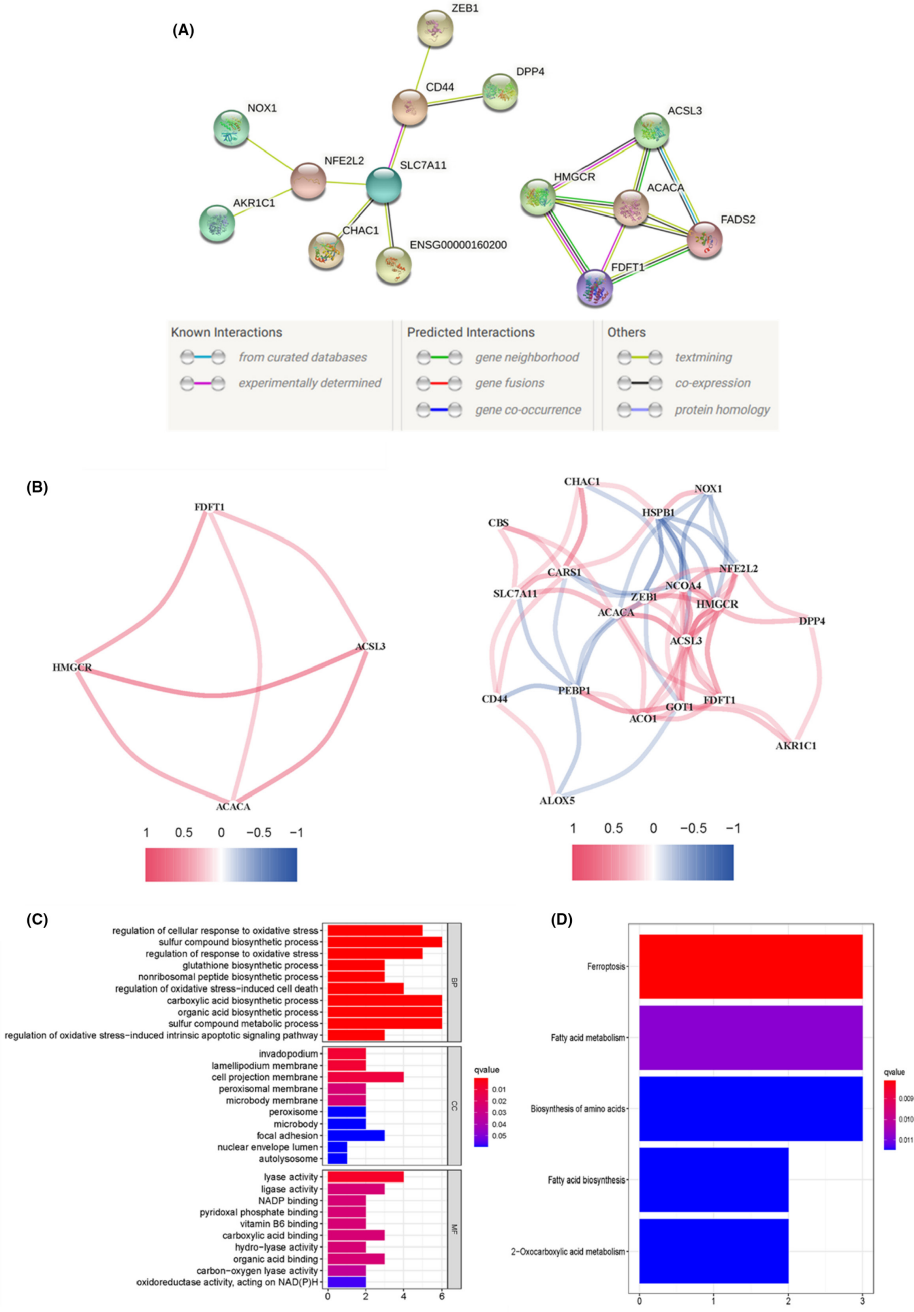
Protein–protein interaction (PPI) network and enrichment analyses. (A) PPI networks. (B) The correlation analysis results. (C) Gene ontology enrichment analysis. (D) Kyoto Encyclopedia of Genes and Genomes pathway enrichment analysis

Then, the PPI network of prognostic ferroptosis genes was constructed, and Cytoscape software was used to obtain this significant module (Figure 2A). The correlation analysis showed the correlation between the proteins. Cutoff = 0.2 was set as the filter criteria, red represents a positive correlation between proteins, and blue represents a negative correlation (Figure 2B). A total of five genes were identified as hub genes with degrees ≥3.

KEGG pathway enrichment analyses and GO function enrichment analyses revealed that the biological classification of the prognostic ferroptosis‐associated genes was enriched in sulfur compound biosynthetic process, carboxylic acid biosynthetic process, organic acid biosynthetic process, and sulfur compound metabolic process in the term of biological processes. The cell projection membrane was the main change in the cell component. These genes may mainly exert their molecular function in lyase activity (Figure 2C). The top two changes in KEGG were ferroptosis and fatty acid metabolism (Figure 2D).

### 3.3 Expression of the five hub genes and survival analysis

**FIGURE 3:**
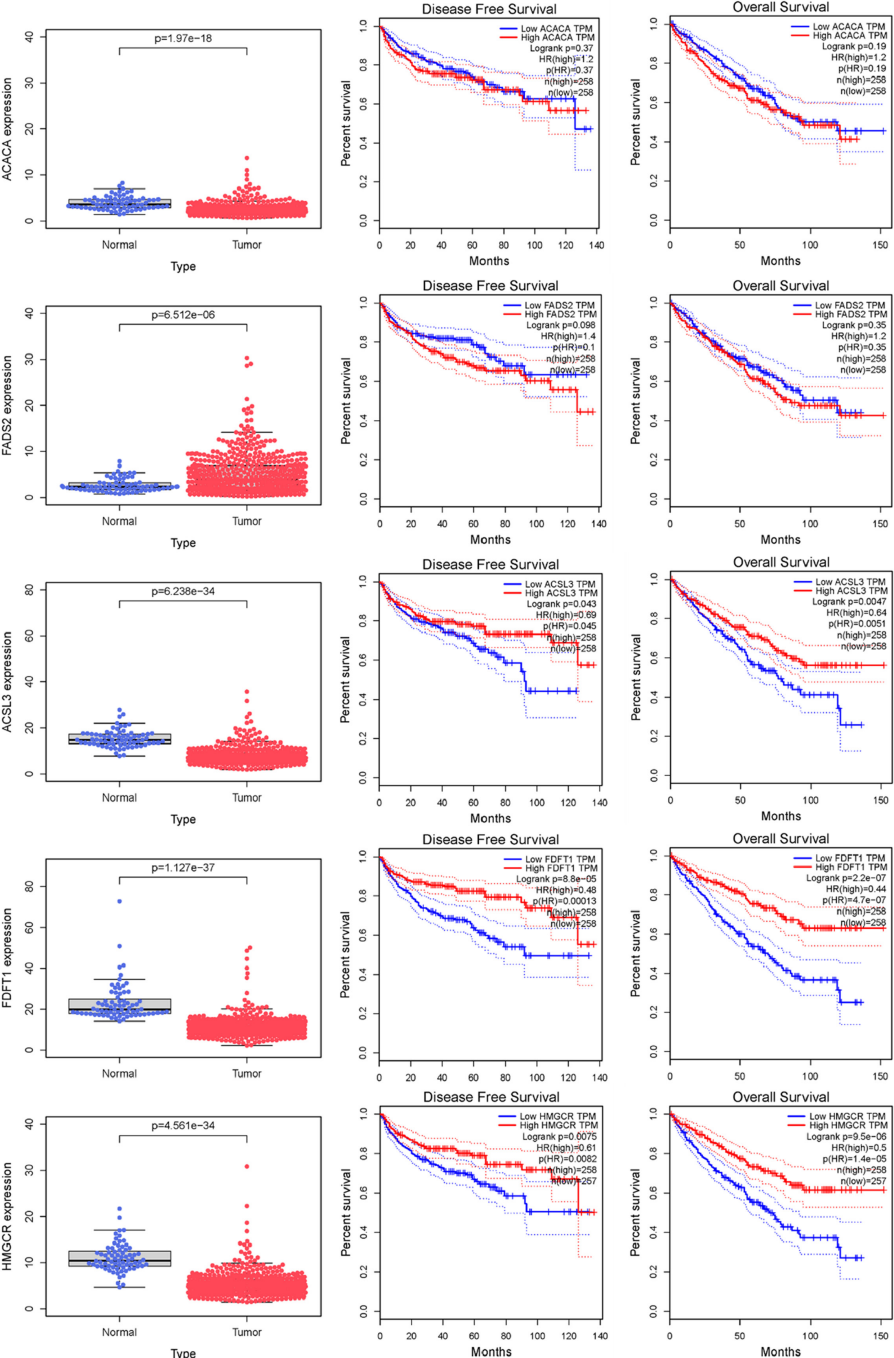
Expression of the five hub genes between tumor and normal tissues, as well as the survival analysis of these genes

The exact expression of the five hub genes between normal tissues and the tumor was demonstrated in box plots accordingly (Figure 3). Although ACACA and FADS2 were differentially expressed between normal tissues and tumors, no significant difference was noted in overall survival (OS) or DFS. Furthermore, it seems that tumor tissues have low expression of ACSL3, FDFT1, and HMGCR, and the high expression of ACSL3, FDFT1, and HMGCR patients was shown to be significantly associated with better OS and DFS, which indicated the suppression roles in carcinogenesis.

### 3.4 FDFT1 overexpression may promote ferroptosis via the AKT signaling pathway

**FIGURE 4:**
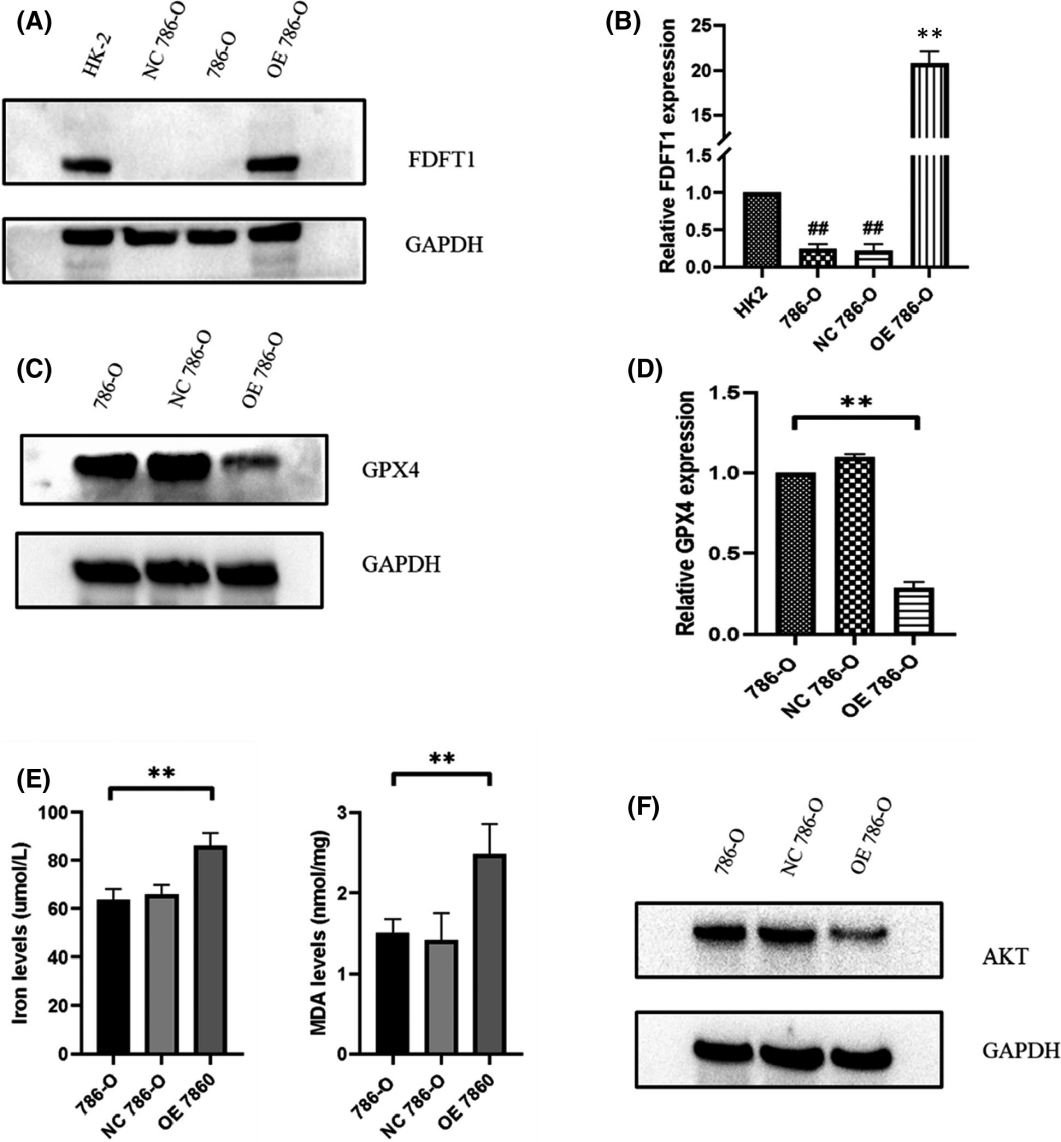
Farnesyl‐diphosphate farnesyltransferase (FDFT1) expression levels and the influence of FDFT1 upregulation on ferroptosis in 786‐O cells. (A, B) Identification of the differentially expressed FDFT1 gene between the normal tissue cell line HK‐2 and the KIRC cell Line 786‐O. (C, D) FDFT1 overexpression decreased the expression of GPX4. (E) Ferrous iron and MDA levels under altered FDFT1 expression. (F) FDFT1 overexpression decreased the expression of AKT (**OE FDFT1 786‐O cells vs. 786‐O and NC 786‐O cells, **p < 0.01; ^##^786‐O and NC 786‐O cells vs. HK‐2 cells, ^##^ *p* < 0.01)

To determine whether our hub genes exerted their functions as we predicted by the bioinformatics method, we selected FDFT1 for further study. Western blot and qPCR results showed that FDFT1 was downregulated in the KIRC cell Line 786‐O (Figure 4A,B). We further found that the overexpression of FDFT1 by plasmid transfection could downregulate the protein and mRNA levels of the ferroptosis biomarker GPX4 (Figure 4C,D), whose low expression level was associated with increased sensitivity to ferroptosis.^15^, ^16^ Iron assays and the lipid peroxidation product MDA were used to examine accumulation in FDFT1‐upregulated 786‐O cells (Figure 4E). These results suggested that FDFT1 participated in the metabolic processes of lipid metabolism and the biological process of ferroptosis.

The Akt signaling pathway has been proven to participate in a series of biological mechanisms.^17^ Thus, we examined whether the Akt signaling pathway was involved in FDFT1‐regulated 786‐O cell development. Intriguingly, the Western blot results revealed that the Akt expression level was decreased when the FDFT1 level was upregulated (Figure 4F).

### 3.5 Upregulated expression of FDFT1 inhibits 786‐O cell proliferation, migration, and invasion

**FIGURE 5:**
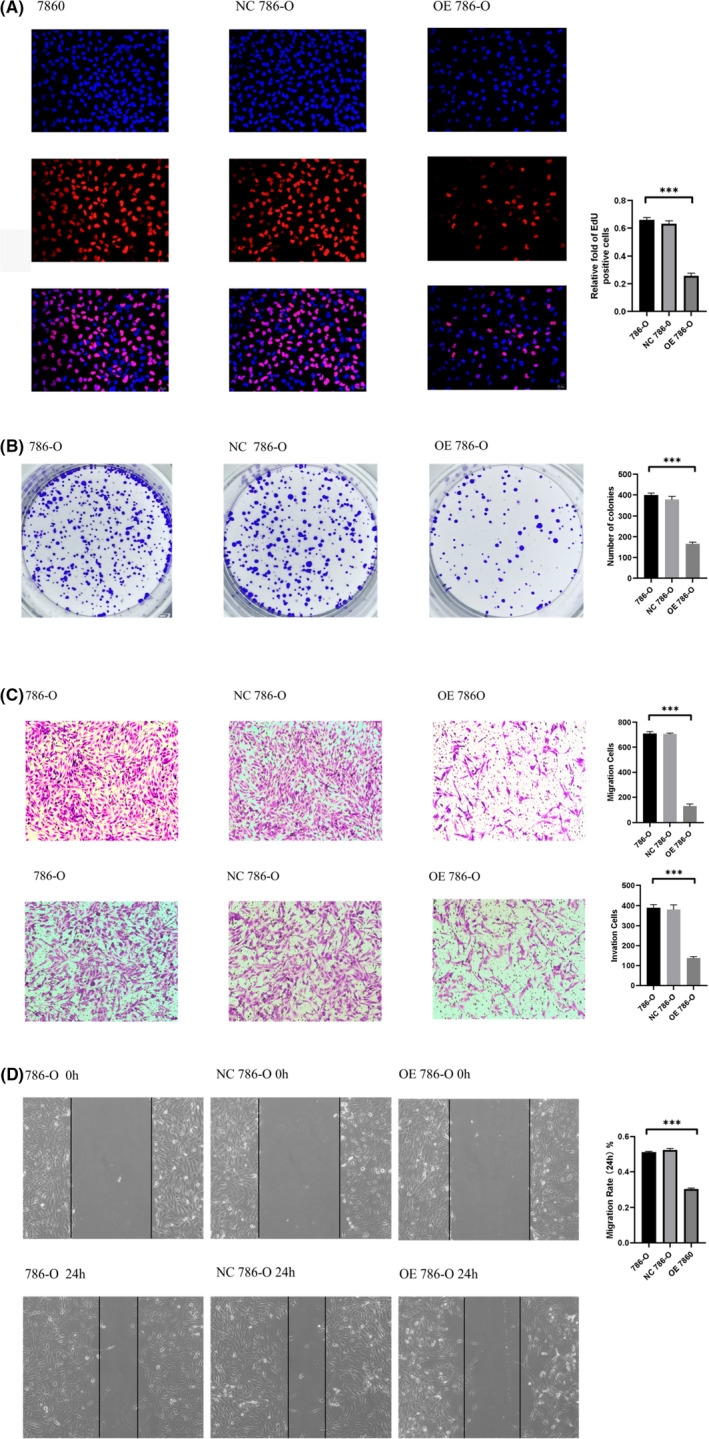
Altered farnesyl‐diphosphate farnesyltransferase (FDFT1) expression in 786‐O cell proliferation, migration, and invasion abilities. (A) EdU assay in each group. (B) Colony formation assay. (C) Transwell migration and invasion assay. (D) Wound healing assay (***OE FDFT1 786‐O cells vs. 786‐O and NC 786‐O, ***p < 0.001)

We focused on regulating FDFT1 expression to evaluate the potential mechanism of FDFT1 in the progression of KIRC. Upregulated expression of FDFT1 was realized by plasmid transfection. The transfection efficiency was examined by qPCR and western blot analysis. Our results illustrated that overexpression of FDFT1 remarkably attenuated 786‐O cell proliferation according to the EdU assay (Figure 5A). Similarly, our colony formation assay also revealed that the cell proliferation ability was significantly alleviated by the overexpression of FDFT1 (Figure 5B). The invasion and migration capacity after upregulation of FDFT1 and the corresponding NC plasmid was investigated by Transwell analysis (Figure 5C) and wound healing assays (Figure 5D). The results showed that in the FDFT1 overexpression group, cell migration and invasion were hampered compared with the corresponding NC group, indicating the potential cancer suppression ability.

## 4 DISCUSSION

Cholesterol is an important substance indispensable to human tissue cells and is involved in various aspects of human health and disease. It not only participates in the formation of cell membranes, but it is also the basic material of bile acid and vitamin D. It is generally believed that the accumulation of cholesterol is the culprit in the development of cardiovascular and cerebrovascular diseases in the elderly. However, multiple lines of evidence have identified that disordered cholesterol homeostasis could be a carcinogenic factor, thus leading to cancer progression, especially intracellular cholesterol levels.^18^ FDFT1 has been identified as the key enzyme for the synthesis of cholesterol. Furthermore, researchers have indicated that downregulated FDFT1 expression in kidney tumors could become a potential biomarker for kidney cancer diagnosis or therapies.^19^

Ferroptosis is a promising type of cell death pathway that is considered an essential link in the tumor suppression mechanism.^20^ Eliminating the key nutrient‐deficient malignant cells in the environment or cells that were damaged by infection or ambient stress led to the depression of tumorigenesis.^21^ In our study, the KEGG analysis results demonstrated that FDFT1 was mainly enriched in the ferroptosis pathway. Hence, we further investigated the role of FDFT1 in ferroptosis in KIRC. Our study provided evidence that FDFT1 overexpression could regulate GPX4 expression. The related indicators of ferroptosis, such as ferrous iron and MDA levels, in the KIRC cell Line 786‐O were also evaluated to determine whether FDFT1 was associated with ferroptosis. Our results indicated that FDFT1 may play a pivotal role in the development of KIRC by regulating ferroptosis.

From a clinical perspective, TCGA datasets showed that high FDFT1 expression was associated with better prognosis in KIRC patients. Previous studies addressed that the inhibition of FDFT1 significantly inhibited prostate cancer cell proliferation, reflecting the antitumor effects in prostate cancer development and its aggressive phenotypes by suppression of FDFT1 expression.^22^ The important role of FDFT1 was also illustrated in ovarian cancer,^23^ colorectal cancer,^24^ and lung cancer.^25^ However, few studies have focused on FDFT1 in the field of KIRC research. Therefore, to further elucidate the function of FDFT1 in the physiopathologic mechanism of KIRC, we paid special attention to the biological behaviors of KIRC cells. Intriguingly, our study validated that upregulated expression of FDFT1 in 786‐O cells was correlated with poor proliferation, migration, and invasion abilities. This may, in other words, be related to FDFT1 overexpression inhibiting malignant progression and improving patient prognosis in KIRC.

Akt is a serine/threonine kinase that can modulate many biochemical mechanisms once it is activated by a range of signals. Molecules that participate in cellular survival, proliferation, migration, metabolism, and angiogenesis have been reported to be downstream targets of Akt. There is growing evidence that Akt itself and its downstream effectors are highly related to tumor development in various types of cancers.^26^ A study on cholesterol deprivation in prostate cancer cells and xenografts found that tumor growth was suppressed and phosphorylation levels of Akt were reduced in the presence of alterations in lipid raft cholesterol content.^27^ In addition, FDFT1 is one of the key enzymes for the synthesis of cholesterol; therefore, the study of FDFT1 in cancer development is of great importance in cancer management. In our study, the overexpression of FDFT1 decreased the level of Akt expression, which was consistent with a previous study in colorectal cancer.^24^

Unfortunately, we failed to illustrate the specific molecular mechanisms of FDFT1 in the process of oncogenesis and its role in regulating ferroptosis via the Akt pathway. Our team will focus on studying the underlying mechanism and how FDFT1 exerts its antitumor effects in regulating cholesterol production and ferroptosis in the future. The protective mechanisms by which FDFT1 affects KIRC still need to be further explored.

## 5 CONCLUSION

In this study, we found that high expression of ACSL3, FDFT1, and HMGCR was associated with better OS and DFS. FDFT1 overexpression inhibited KIRC cell proliferation, migration, and invasion, and the ferroptosis and AKT signaling pathways were involved.

## CONFLICT OF INTEREST

No conflict of interest exist in the submission of this manuscript, and the manuscript has been approved by all authors for publication.

## ETHICS STATEMENT

This study was assessed and approved by the Ethics Committee for Animal Experiments of the Second Affiliated Hospital of Nanchang University.

## AUTHORS' CONTRIBUTIONS

This work was designed by Honglin Hu. Ruizhen Huang, Chiyu Zhang, Xing Wang, Xin Zou, Zhengjie Xiang, TL, BG, and ZW performed the whole study. RH, CZ, and XW analyzed and interpreted the data acquired by the bioinformatics method. RH drafted the paper and was responsible for the paper revision.

## Data Availability

The datasets used and/or analyzed during the current study are available from the corresponding author on reasonable request.

## References

[1] Siegel RL , Miller KD , Jemal A . Cancer statistics, 2018. CA Cancer J Clin. 2018;68:7‐30. doi:10.3322/caac.21442 29313949

[2] Moch H , Cubilla AL , Humphrey PA , Reuter VE , Ulbright TM . The 2016 WHO classification of Tumours of the urinary system and male genital organs‐part a: renal, penile, and testicular Tumours. Eur Urol. 2016;70:93‐105. doi:10.1016/j.eururo.2016.02.029 26935559

[3] Ghatalia P , Gordetsky J , Kuo F , et al. Prognostic impact of immune gene expression signature and tumor infiltrating immune cells in localized clear cell renal cell carcinoma. J Immunother Cancer. 2019;7:139. doi:10.1186/s40425-019-0621-1 31138299 PMC6540413

[4] Rao A , Wiggins C , Lauer RC . Survival outcomes for advanced kidney cancer patients in the era of targeted therapies. Ann Transl Med. 2018;6:165. doi:10.21037/atm.2018.04.44 29911113 PMC5985277

[5] Dixon SJ . Ferroptosis: bug or feature? Immunol Rev. 2017;277:150‐157. doi:10.1111/imr.12533 28462529

[6] Jiang L , Kon N , Li T , et al. Ferroptosis as a p53‐mediated activity during tumour suppression. Nature. 2015;520:57‐62. doi:10.1038/nature14344 25799988 PMC4455927

[7] Yu Y , Xie Y , Cao L , et al. The ferroptosis inducer erastin enhances sensitivity of acute myeloid leukemia cells to chemotherapeutic agents. Mol Cell Oncol. 2015;2:e1054549. doi:10.1080/23723556.2015.1054549 27308510 PMC4905356

[8] Nie J , Shan D , Li S , et al. A novel ferroptosis related gene signature for prognosis prediction in patients with colon cancer. Front Oncol. 2021;11:654076. doi:10.3389/fonc.2021.654076 34046350 PMC8144717

[9] Park JM , Mau CZ , Chen YC , et al. A case‐control study in Taiwanese cohort and meta‐analysis of serum ferritin in pancreatic cancer. Sci Rep. 2021;11:21242. doi:10.1038/s41598-021-00650-7 34711879 PMC8553768

[10] Toth C , Funke S , Nitsche V , et al. The role of apoptosis repressor with a CARD domain (ARC) in the therapeutic resistance of renal cell carcinoma (RCC): the crucial role of ARC in the inhibition of extrinsic and intrinsic apoptotic signalling. Cell Commun Signal. 2017;15:16. doi:10.1186/s12964-017-0170-5 28464919 PMC5414156

[11] Yang WS , SriRamaratnam R , Welsch ME , et al. Regulation of ferroptotic cancer cell death by GPX4. Cell. 2014;156:317‐331. doi:10.1016/j.cell.2013.12.010 24439385 PMC4076414

[12] Miess H , Dankworth B , Gouw AM , et al. The glutathione redox system is essential to prevent ferroptosis caused by impaired lipid metabolism in clear cell renal cell carcinoma. Oncogene. 2018;37:5435‐5450. doi:10.1038/s41388-018-0315-z 29872221 PMC6173300

[13] Liang JY , Wang DS , Lin HC , et al. A novel ferroptosis‐related gene signature for overall survival prediction in patients with hepatocellular carcinoma. Int J Biol Sci. 2020;16:2430‐2441. doi:10.7150/ijbs.45050 32760210 PMC7378635

[14] Tang Z , Li C , Kang B , Gao G , Li C , Zhang Z . GEPIA: a web server for cancer and normal gene expression profiling and interactive analyses. Nucleic Acids Res. 2017;45:W98‐W102. doi:10.1093/nar/gkx247 28407145 PMC5570223

[15] Seibt TM , Proneth B , Conrad M . Role of GPX4 in ferroptosis and its pharmacological implication. Free Radic Biol Med. 2019;133:144‐152. doi:10.1016/j.freeradbiomed.2018.09.014 30219704

[16] Zou Y , Palte MJ , Deik AA , et al. A GPX4‐dependent cancer cell state underlies the clear‐cell morphology and confers sensitivity to ferroptosis. Nat Commun. 2019;10:1617. doi:10.1038/s41467-019-09277-9 30962421 PMC6453886

[17] Fresno Vara JA et al. PI3K/Akt signalling pathway and cancer. Cancer Treat Rev. 2004;30:193‐204. doi:10.1016/j.ctrv.2003.07.007 15023437

[18] Kuzu OF , Noory MA , Robertson GP . The role of cholesterol in cancer. Cancer Res. 2016;76:2063‐2070. doi:10.1158/0008-5472.CAN-15-2613 27197250 PMC5813477

[19] Tuzmen S et al. Characterization of farnesyl diphosphate farnesyl transferase 1 (FDFT1) expression in cancer. Per Med. 2019;16:51‐65. doi:10.2217/pme-2016-0058 30468409

[20] Su Y , Zhao B , Zhou L , et al. Ferroptosis, a novel pharmacological mechanism of anti‐cancer drugs. Cancer Lett. 2020;483:127‐136. doi:10.1016/j.canlet.2020.02.015 32067993

[21] Fearnhead HO , Vandenabeele P , Van den Berghe T . How do we fit ferroptosis in the family of regulated cell death? Cell Death Differ. 2017;24:1991‐1998. doi:10.1038/cdd.2017.149 28984871 PMC5686356

[22] Fukuma Y , Matsui H , Koike H , et al. Role of squalene synthase in prostate cancer risk and the biological aggressiveness of human prostate cancer. Prostate Cancer Prostatic Dis. 2012;15:339‐345. doi:10.1038/pcan.2012.14 22546838

[23] Zheng L , Li L , Lu Y , Jiang F , Yang XA . SREBP2 contributes to cisplatin resistance in ovarian cancer cells. Exp Biol Med (Maywood). 2018;243:655‐662. doi:10.1177/1535370218760283 29466876 PMC6582395

[24] Weng ML , Chen WK , Chen XY , et al. Fasting inhibits aerobic glycolysis and proliferation in colorectal cancer via the Fdft1‐mediated AKT/mTOR/HIF1alpha pathway suppression. Nat Commun. 2020;11:1869. doi:10.1038/s41467-020-15795-8 32313017 PMC7170903

[25] Dehghani M , Samani Z , Abidi H , et al. Relationship of SNP rs2645429 in farnesyl‐diphosphate farnesyltransferase 1 gene promoter with susceptibility to lung cancer. Int J Genomics. 2018;2018:4863757. doi:10.1155/2018/4863757 29765975 PMC5885393

[26] Revathidevi S , Munirajan AK . Akt in cancer: mediator and more. Semin Cancer Biol. 2019;59:80‐91. doi:10.1016/j.semcancer.2019.06.002 31173856

[27] Zhuang L , Kim J , Adam RM , Solomon KR , Freeman MR . Cholesterol targeting alters lipid raft composition and cell survival in prostate cancer cells and xenografts. J Clin Invest. 2005;115:959‐968. doi:10.1172/JCI19935 15776112 PMC1064980

